# Application of linker technique to trap transiently interacting protein complexes for structural studies

**DOI:** 10.14440/jbm.2016.81

**Published:** 2016-01-28

**Authors:** Vishnu Priyanka Reddy Chichili, Veerendra Kumar, J. Sivaraman

**Affiliations:** Department of Biological Sciences, National University of Singapore, Singapore, 117543

**Keywords:** protein-protein interactions, transient interactions, glycine-rich linker

## Abstract

Protein-protein interactions are key events controlling several biological processes. We have developed and employed a method to trap transiently interacting protein complexes for structural studies using glycine-rich linkers to fuse interacting partners, one of which is unstructured. Initial steps involve isothermal titration calorimetry to identify the minimum binding region of the unstructured protein in its interaction with its stable binding partner. This is followed by computational analysis to identify the approximate site of the interaction and to design an appropriate linker length. Subsequently, fused constructs are generated and characterized using size exclusion chromatography and dynamic light scattering experiments. The structure of the chimeric protein is then solved by crystallization, and validated both *in vitro* and *in vivo* by substituting key interacting residues of the full length, unlinked proteins with alanine. This protocol offers the opportunity to study crucial and currently unattainable transient protein interactions involved in various biological processes.

## BACKGROUND

Molecular recognition lies at the heart of all biological processes. Interactions between two proteins can be characterized in terms of binding affinity, which describes how tightly two binding partners interact. The binding process is regarded as an equilibrium, resulting from a balance between association and dissociation events [1]. Protein-protein binding affinity can be influenced by various non-covalent intermolecular interactions, such as hydrogen bonds, electrostatic, hydrophobic and Van der Waals forces, as well as by macromolecular crowding caused by high concentrations of macromolecules other than the binding partner(s) [2]. Protein complexes with a low dissociation constant (~ nM) are regarded as high affinity protein complexes, such as interactions between antigen and antibody complexes, whereas protein complexes with a high dissociation constant have a low affinity, such as protein complexes involved in intracellular signaling. In biological systems, these associations are usually reversible, but irreversible covalent bonding can also be observed [3-5].

Since the realization that many diseases stem from abnormalities in protein-protein interactions, it has become imperative to elucidate how changes in binding influence disease progression in order to develop appropriate therapeutics. While stable, high affinity protein-protein complexes can be generated following well-established procedures [6,7], most intracellular signaling events are transient, adopting a ‘hit-and-run’ strategy that makes it difficult to trap the interaction for structure determination. These transient interactions can be classified into two types: (1) interactions where binding partners have an ordered structure or (2) interactions where one or more of the binding partners only gains secondary structure upon binding.

In recent years, a large number of proteins in the eukaryotic proteome have been determined to be intrinsically unstructured proteins (IUPs) [8-11]; i.e., they contain no or very little well-defined structure and lack a compact globular fold. IUPs are involved in transcriptional regulation, cellular signaling, cell cycle control, endocytosis, replication, and biogenesis of the cytoskeleton [12,13], and they can bind to different proteins using consistent or different interfaces [14]. Unfolded proteins have been reported to fold or attain secondary structure upon binding with their interacting partner(s) [15,16], and studies note that these disordered regions that undergo a transition into order are generally shorter regions [17], in some cases, less than 30 residues [17]. Nevertheless, this highly flexible state of an IUP is fundamental to their biological role, and allows them to bind multiple partners and adopt different conformations. The intrinsic flexibility of these proteins also confers several functional advantages, such as specificity without excessive binding strength, increased speed of interaction, and binding promiscuity, all highly desirable for signaling and regulatory processes [17].

**Figure 1.**
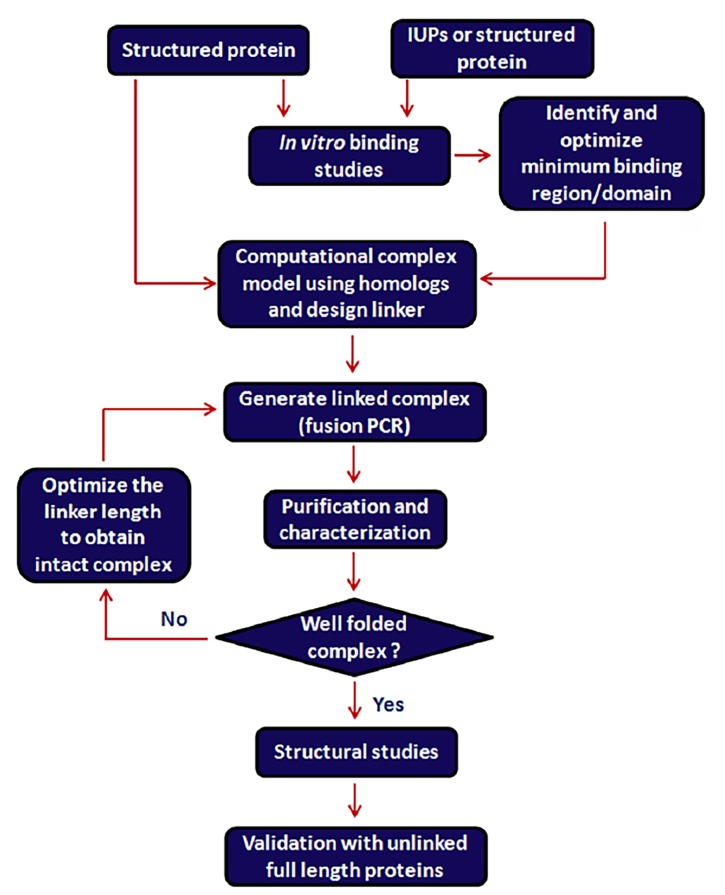
Flowchart for trapping transiently interacting protein complexes using linker technique for structural studies.

Methods such as X-ray crystallography, NMR spectroscopy, and electron microscopy require stable protein complexes. Thus, in order to understand the nature of transient and unstructured protein-protein interactions, a method is required to trap them into a stable conformation. Here, we present a protocol to trap transiently interacting protein complexes, for which one of the binding partners is unstructured (**Fig. 1**). We employed a flexible glycine (Gly) linker to fuse the two interacting partners. Being highly flexible, the poly-Gly linker will not impose any spatial restriction on the mobility of the linked proteins. The presence of the linker increases the proximity of the two proteins of interest and retains the interaction. Following a review of the available literature [18], we devised a method to design and produce a chimeric protein construct using a suitable Gly or Gly-rich linker, and proceeded to test this method using an interaction that was previously unable to be solved by co-crystallization [19]: Calmodulin (CaM) binding with two of its intrinsically disordered, neuron-specific substrate proteins, Neuromodulin (Nm) and Neurogranin (Ng). Initial attempts to co-crystallize CaM with the IQ motifs of Nm and Ng did not yield complex crystals despite having strong evidences for the complex formation. Employing this method we generated stable complexes of CaM with the IQ motifs of Nm and Ng IQ using a flexible Gly linker, crystallized and solved the structures, and validated the results with unlinked full-length proteins and alanine substitution mutagenesis.

For the first time, here we present a detailed protocol for the design and validation of Gly-linkers to trap the transiently interacting protein complexes, where one of the binding partners is intrinsically disordered. When a protein complex is first targeted for structural study, initial attempts are made to determine the affinity and stability of the complex using various biophysical techniques such as isothermal titration calorimetry (ITC), surface plasmon resonance (SPR) and other fluorescence methods. Often, working with the full-length unstructured protein is troublesome and can be replaced with a peptide comprising the minimum binding region (MBR) to mimic the binding of the full-length proteins. The MBR can be determined using co-immunoprecipitation, gel-shift assays and biophysical binding studies. In the present case, the MBRs of Nm and Ng were previously determined to be the IQ motif [20,21]. We thus designed two different length peptides (19 and 24 amino acids) from Nm and Ng and assessed their binding affinities towards CaM using ITC.

If the specific site of the interaction is known, a Gly or Gly-rich linker of an appropriate length can be designed to facilitate positioning of the MBR peptide within proximity of the binding region of the stable protein; this can be achieved with the knowledge that each glycine residue corresponds to a length of ~3.8 Å [22]. If the site of the interaction is unknown, various studies such as mutagenesis, docking experiments, limited proteolysis and Hydrogen/Deuterium (H/D) exchange experiments can be employed and various linker lengths can be tested to identify the appropriate length that facilitates an interaction. In the present case, we used DeepView analysis of a known CaM-peptide complex, CaM-myosin V IQ motif peptide complex (PDB code: 2IX7), to estimate the approximate site of the interaction for Nm and Ng with CaM. Thus, using literature and computational analyses, we identified a (Gly)_5_ linker as sufficient to link the Nm/Ng MBR to the C-terminus of CaM.

A three-step fusion PCR procedure was then employed to link the two proteins, where the sequence for the linker is incorporated into the reverse primer of the CaM gene and the forward primer of Nm/Ng IQ motif gene, with fusion performed in the next round of PCR. The fused constructs are expressed in *E. coli* and the proteins are then purified using Ni-NTA affinity chromatography and the chimeric proteins characterized by size-exclusion chromatography (SEC) and dynamic light scattering (DLS). Further analytical ultra-centrifugation (AUC) and circular dichroism (CD) can also be performed to verify the presence of a well-folded intact complex. While we describe here the use of hanging drop vapor diffusion for crystallization of the linked complexes, sitting drop and under oil methods can also be used. Finally, the structural results of the complex obtained with the chimeric protein derived by linking the binding partners was validated by mutating various key interacting residues identified from the linked complex in full-length unlinked proteins/domains *in vitro* and *in vivo*. We validated our structural findings using ITC and relevant electrophysiological experiments *in vivo*.

## MATERIALS

### Reagents

• LB medium (Merck, cat. # 71753-6)
• LB agar (Sigma-Aldrich, cat. # L2897)
• CaCl_2_ (purity > 99.8%; Fluka, cat. # 21075)
• Agarose (Analytical grade; Promega, cat. # V3125)
• Seleno-L-methionine (purity > 98%; Sigma-Aldrich, cat. # S3132)
• Ampicillin (purity > 96%; Sigma-Aldrich, cat. # A9393)
• Isopropyl-β -D-thiogalactoside, IPTG (GoldBio; cat. # 12481C200)
• Tris base (purity > 99.8%; Fluka, cat. # 252859)
• Imidazole (purity > 99%; Sigma-Aldrich, cat. # I5513)
• HCl (purity 37%; Sigma-Aldrich, cat. # 320331)
• NaCl (purity > 98%; Sigma-Aldrich, cat. # S3014)
• EGTA (purity > 97%; Sigma-Aldrich, cat. # E3889)
• Glycerol (purity > 99%; Sigma-Aldrich, cat. # G5516)
• Triton X-100 (Sigma-Aldrich, cat. # T8787)
• Ni-NTA (Qiagen, cat. # 30230)
• Coomassie protein assay reagent (ThermoFisher Scientific, cat. # 1856209)
• Non-ionic detergent or methanol (for washing the ITC system)
• Distilled water
• Commercially available crystallization screens from Hampton Research, Qiagen (JCSG and PACT suite), Jena Biosciences and Emerald Biosystems
• Kao and Michayluk vitamin solution (Sigma-Aldrich, cat. # K3129)

### Recipes

Different lengths of Nm and Ng IQ motif peptides were commercially synthesized and obtained from GL BiochemLtd (Shanghai, China). Stock solutions of 1 mM of Nm/Ng IQ peptides were prepared by dissolving the appropriate amount of peptide in 20 mM imidazole pH 8.0, 100 mM NaCl and 2 mM EGTA (Buffer A). After mixing, the peptide stock solutions were stored at 4°C to ensure complete peptide solubility. The choice of buffer is based on obtaining maximum solubility of the peptides. **NOTE:** Synthetically derived peptides should be verified for solubility when dissolved in a buffer of interest. Sometimes preparing high concentrations of peptide stock solution may result in precipitation. Lower concentrations of stock solutions can be prepared to avoid this problem.

• TAE buffer: Tris-acetate-EDTA buffer is used for preparing agarose gels and for electrophoresis.
• Double digestion: *Nde*I and *Xho* restriction enzymes were from New England Biolabs, UK. Double digestion was performed using these enzymes according to manufacturer’s instructions.
• T4 DNA ligase: Double digested vector and insert were ligated using T4 DNA ligase (Fermentas) using manufacturer’s instructions.
• Gel extraction kit: PCR products were purified using the GeneAll kit (GeneAll Biotechnology, Korea). Other commercially available kits can also be used.
• Plasmid preparation kit: Plasmids purified using the Qiagen Plasmid Prep Kit. Other commercially available kits can also be used.
• 0.1 M CaCl_2_: For 50 ml of solution, weigh 0.55 g of CaCl_2_, add 45 ml of water, dissolve it completely and bring the total volume to 50 ml. Sterilize using 0.22 µm filters.
• BL21 (DE3)/DH5α/DL41 competent cells preparation: Take 2 ml of overnight-grown BL21 (DE3) or DH5α cultures and inoculate 50 ml of LB medium. Allow the cells to grow at 37°C until the OD_600_ reaches 0.4–0.5. Place the cells on ice for 5 min, and then spin at 1200 *g* for 10 min. Remove the supernatant, and resuspend the pellet in 40 ml of 0.1 M CaCl_2_. Incubate the resuspended pellet on ice for 45 min and then spin again at 1200 *g* for 10 min. Remove the supernatant and resuspend the pellet in 2.5 ml of 2 ml of 0.1 M CaCl_2_ mixed with 0.5 ml of autoclaved 100% glycerol (Final solution). Dispense 50 µl of the resuspended pellet in 1.7 ml microfuge tubes and snap freeze using liquid nitrogen for long term storage. CAUTION: Liquid nitrogen is -196°C. Wear cryoprotective gloves and a face mask while handing liquid nitrogen.
• Transformation: Use BL21 (DE3)/DH5α/DL41 competent cells for transformation. Thaw one microfuge tube of cells on ice for 10 min. Add 1 µl of the required plasmid for BL21 (DE3) transformation and 10 µl of reaction mixture for DH5α transformation. Incubate the cells on ice for 30 min. Heat shock the cells for 90 s at 42°C and then return the cells to ice for 2 min. Add 150 µl of LB medium and transfer the cells to a shaker for 1 h at 37°C at 180 rpm. Plate the entire cell suspension onto LB-Agar plates supplemented with 100 µg/ml of ampicillin.
• LB medium: Measure 25 g of LB broth and mix with 1 L of deionized water in 2.8 L flasks. Autoclave this medium at 121°C for 20 min.
• LB-Agar plates: Measure 20 g of LB-agar and mix with 500 ml of deionized water. Autoclave the mixture at 121°C for 20 min and then allow the solution cool to less than 50°C. Add 500 µl of 100 mg/ml of ampicillin and mix well. Pour approximately 20 ml of this mixture in each petri plate and stored at 4°C for long-term storage.
• Tris-HCl buffer (1.0 M pH 7.5): For a 500 ml solution, add 61 g of Tris base to 400 ml of water, and adjust the pH to 7.4 with 1 M HCl. Bring the total volume to 500 ml.
• Imidazole buffer (1.0 M pH 8.0): For a 500 ml solution, add 34.3 g of imidazole to 400 ml of water, and adjust the pH to 8.0 with 1 M HCl. Bring the total volume to 500 ml.
• NaCl solution (4 M): For a 500 ml solution, add 117 g of NaCl to 450 ml of water, dissolve it completely, and then bring the total volume to 500 ml.
• EGTA solution (0.5 M pH 8.0): For a 100 ml solution, add 38 g of EGTA to 80 ml of water, and adjust the pH to 8.0 with 1 M HCl. Bring the total volume to 100 ml.
• Lysis buffer solution (50 mM Tris pH 7.4, 200 mM NaCl, 5% glycerol, 0.1%TritonX, 5 mM imidazole): For 100 ml of lysis buffer, add 5 ml of 1 M Tris-HCl pH 7.4; 5 ml of 4 M NaCl; 5 ml of glycerol; 100 µl of TritonX-100; 500 µl of 1 M imidazole; and 84.4 ml of water. **NOTE**: Prepare fresh before use.
• Superdex 75 running buffer or Buffer A (20 mM imidazole pH 8.0, 100 mM NaCl and 2 mM EGTA): For 500 ml of Buffer A, add 10 ml of 1 M imidazole pH 8.0; 12.5 ml of 4 M NaCl; 2 ml of 0.5 M EGTA pH 8.0; and 475.5 ml of water. **NOTE**: Prepare fresh before use.
• LeMaster medium [23]: First, prepare a homogenous mixture of the following reagents and store at -20°C: 5 g of alanine, 5.8 g of arginine HCl, 8 g of aspartic acid, 0.3 g of cysteine, 6.7 g of glutamic acid, 3.3 g of glutamine, 5.4 g of glycine, 0.6 g of histidine, 2.3 g of leucine, 4.2 g of lysine HCl, 1.3 g phenylalanine, 1.0 g of proline, 20.8 g of serine, 2.3 g of threonine, 1.7 g of tyrosine, 2.3 g of valine, 5.0 g of adenine, 6.7 g of guanosine, 1.7 g of thymine, 5.0 g of uracil, 15.0 g of sodium acetate, 15.0 g of succininc acid, 7.5 g of ammonium chloride, 10.8 g of sodium hydroxide, 105.0 g of K_2_HPO_4_ dibasic. Second, to prepare the medium, combine 24.1 g of the homogenous mixture with 1 L of deionized water and autoclave at 121°C for 20 min. Third, dissolve 10 g of D-glucose, 0.25 g of MgSO_4_.7H_2_0 and 4.18 mg of FeSO_4_.7H_2_0 in 90 ml of deionized water, add 8.3 µl of concentrated H_2_SO_4_ and 10 ml of Kao and Michayluk vitamin solution and sterilize by filtration. Add this third mixture to the autoclaved medium. Add 25 mg of seleno-L-methionine and 1 ml of 100 mg/ml of ampicillin prior to inoculation. This corresponds to 1.1 L of LeMaster medium. **NOTE**: Prepare fresh before use. CAUTION: Seleno-L-Methionine is highly toxic. Handle with great precaution and wear gloves and a mask. Prior to inoculation, plasmids containing the gene of interest should be transformed into methionine auxotrophic strain (DL41) for seleno-L-methionine incorporated protein production.
• Coomassie protein assay reagent: Coomassie protein assay was used to measure protein concentration. Homogenously mix 0.5 ml of water and 0.5 ml of reagent and use it as blank. For each sample, add 1 µl of protein solution to this blank solution and mix well. Measure the absorbance of the sample at 595 nm using the blank as a reference. **NOTE**: If the concentration of the protein is above 5 mg/ml, it is advisable to dilute the protein for accurate measurement of protein concentration.
• Optimization of crystallization conditions: Once the initial crystallization conditions are identified from commercial screens, further optimize this condition to obtain the best diffraction-quality crystals. In the present case, we obtained crystals in magnesium acetate, PEG3350 and sodium citrate tribasic conditions. Hence, the following stock solutions are prepared: 1.0 M magnesium acetate tetrahydrate (Dissolve 10.7 g of MgAcO in 45 ml of water, and top up to 50 ml), 50% w/v PEG3350 (Dissolve 25 g of PEG3350 in 30 ml of water, using a hot plate if necessary to dissolve completely, and make up to a final volume of 50 ml) and 1.6 M sodium citrate tribasic (Dissolve 23.5 g of sodium citrate tribasic in 40 ml of water and top up to 50 ml). **NOTE**: Prepare fresh before use.

### Equipment

• Isothermal titration calorimeter (VP-ITC, MicroCal LLC) or equivalent
• Origin 7.0 or equivalent data analysis software
• Vacuum pump
• Long-needle syringe (2.5 ml; Hamilton 1002LLSN, Hamilton)
• Stirring injection syringe part (VP-ITC, MicroCal LLC, part no. SYA13022; stirring speed, 300 rpm.)
• Water filtration system (Millipore)
• Kimwipes or equivalent laboratory wipes
• pH meter (Sartorius) or equivalent
• PCR thermocycler (Applied Biosystems) or equivalent
• Autoclave machine
• Petri plates or equivalent
• Weighing Balance (Sartorius) or equivalent
• Pipettes (0.2–2 µl, 1–10 µl, 10–100 µl and 100–1000 µl)
• Sonicator (Sonics) or equivalent
• Avanti J-26 XP centrifuge (Beckman coulter) or equivalent
• Eppendorf centrifuge 5804R or equivalent
• Eppendorf Tabletop centrifuge or equivalent
• AKTA purifier Low-pressure chromatography system (GE Healthcare, cat. # 28-4062-66) or equivalent
• Spectrophotometer (Thermo Scientific) or equivalent
• HiLoad 16/60 Superdex 75 pg column (GE Healthcare, cat. # 28-9893-33)
• Centrifugal filter units (Millipore, cat. # UFC901024) or equivalent
• 1.7 ml microfuge tube (Axygen) or equivalent
• DynaPro for DLS experiments (Protein Solutions) or equivalent
• Dynamics V5 software or equivalent
• 24-well hanging drop crystallization plates (Hampton Research, cat. # HR3-140) or equivalent
• Light microscope with 4× magnification (Olympus) or equivalent
• Mounted cryo loops of appropriate size (Hampton Research) or equivalent
• CrystalCap copper to stalk mounted cryo loops (Hampton Research) or equivalent
• X-ray equipment (Rigaku Raxis V+) or equivalent
• Computers
• DeepView, HKL2000, CCP4, COOT and Pymol software or equivalent

**Table:** 

Reagent	Amount per reaction
Template DNA	10 ng
Primers (P1 and P2 or P3 and P4)	300 nM final concentration
dNTPs	300 µM final concentration
KAPA HiFi DNA Polymerase	1 U
Buffer	As provided by the manufacturer

**Table:** 

Cycle number	Denature	Anneal	Extend
1	95°C, 2 min		
2 (30 times)	98°C, 20 s	52°C, 15 s	72°C, 1 min^b^
3			72°C, 2 min

^b^Change the time according to size, with extension time calculated based on 30 s kb^-1^ for the fragment being amplified.

**Table 1.** Troubleshooting table.

Step	Problem	Possible Reasons	Solution
1.10	Unstable ITC baseline or baseline shift	Sample cell and syringe are dirty	Thoroughly wash the cell and syringe
		Air bubbles in the sample cell	Remove air bubbles. Take care not to introduce bubbles when filling the cell and syringe
2.3	Template PDB not available	No known structures of the binding partners	Search for homologous protein structure of the binding partners and use them as template
		No known homologous protein structures	Perform sequence based 3D structure prediction
3.5	Absence of a PCR product after fusion PCR	Gene fusion did not take place	Recheck the primers and repeat the reaction by slightly varying the annealing temperature
	Multiple PCR products after fusion PCR	The primers may have bound to related sequences elsewhere in the template	Redesign the primers and repeat the reaction
3.8	No colonies observed after transformation	incomplete double digestion	Optimize the duration of restriction double digestion to obtain complete digestion of vector and insert
		Star activity by the restriction digestion enzymes	Optimize the duration of restriction double digestion to reduce star activity
4.5	No protein in elution	Cell culture conditions are not suitable	Try different cell culture conditions by varying the IPTG concentration and temperature for induction and induction period
		Protein is in the insoluble fraction	Use a refolding method or use a solubility tag to make the protein soluble
		No binding with Ni-NTA resin	Use fresh Ni-NTA resin and equilibrate with lysis buffer for a minimum of 1 hr
		Protein eluted away during wash steps	Remove imidazole from wash buffers and repeat the assay
4.7	No protein peak observed in the elution profile	Protein has leaked due to tubing connections.	Check the connections of the loop. Try to reconnect and inject the protein
		UV lamp is not sensitive; this is particularly a concern if the protein concentration is low	Change the UV lamp for better sensitivity
4.7	Protein eluted in void volume	Protein shows higher order oligomerization	Optimize buffer conditions to prevent aggregation
		Binding partners may not be interacting to form an intact complex	Optimize the length of the linker to retain natural interactions between the binding partners
4.8	Protein is not stable	Protein precipitates while concentrating	Optimize buffer conditions to prevent protein precipitation
		Binding partners may not be interacting to form an intact complex	Optimize the length of the linker to retain natural interactions between the binding partners
4.8	Protein concentration does not increase	Protein may have precipitated	Do not concentrate the protein beyond a particular level. Measure the concentration of the protein at frequent intervals
4.13	DLS shows high hydrodynamic radius	Protein tends to aggregate	Optimize buffer conditions to reduce aggregation
5.2	No crystal formation	Protein storage buffer may not be optimal	Try to vary the protein storage buffer composition and re-perform crystallization
		Less number of screening conditions are used	Perform crystallization trials with an increased number of screens
6.4	Absence of PCR product after inverse PCR	Linear PCR product was not amplified	Check the primers and optimize the PCR conditions accordingly

**Figure 2:**
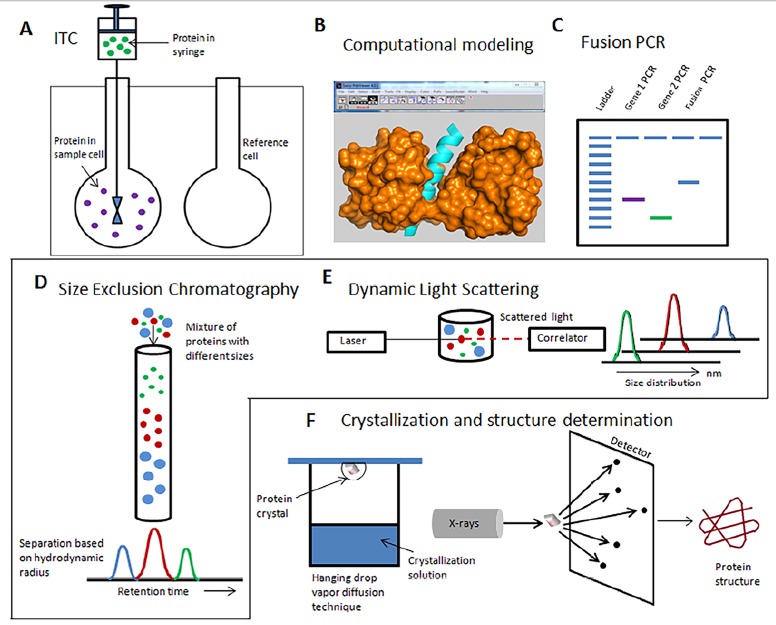
**Schematic representation of key experimental setup required for linker technique to trap transiently interacting protein complexes. A.** Isothermal titration calorimetry (ITC). Reference cell is filled with buffer; the sample cell and syringe are filled with proteins involved in interaction. **B.** Computational analysis. Structurally known homologues protein complexes are used as template to generate model for current protein complex of interest. **C.** Gene fusion. Two genes when fused together using fusion PCR produces a linear PCR product which corresponds to sum of the length of both of the genes. **D.** Size exclusion chromatography (SEC). SEC separates proteins based on their size. Higher molecular weight proteins are eluted earlier than the proteins of low molecular weight. **E.** Dynamic Light Scattering (DLS). DLS is used to characterize the size of proteins by measuring the fluctuations in scattered light intensity by protein molecules. **F.** Structure determination. Hanging drop vapor diffusion method utilizes a droplet consisting of purified protein and crystallization solution being allowed to equilibrate with a larger volume of crystallization solution. Diffraction pattern of crystals are obtained using X-rays and collect a complete dataset for structure determination.

**Figure 3.**
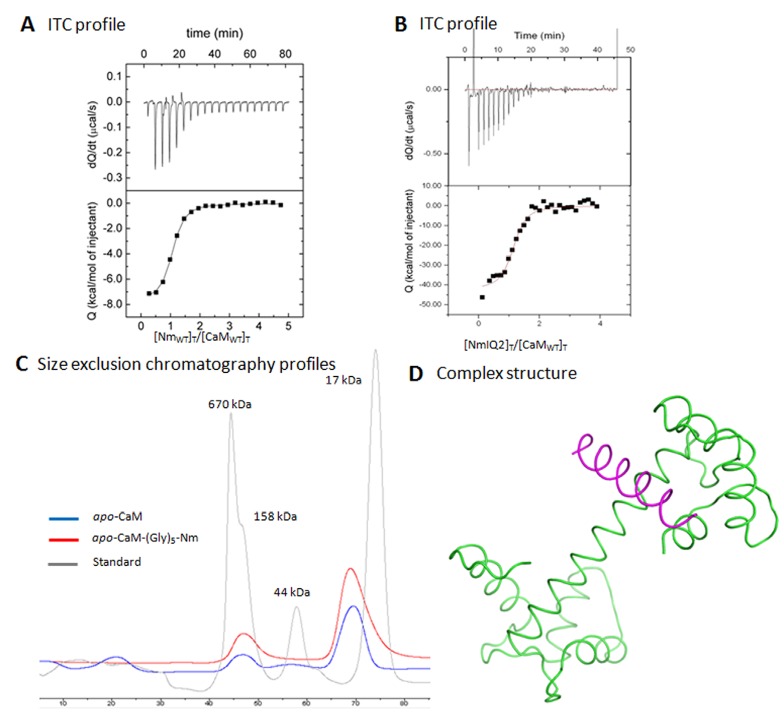
Trapping CaM and IQ motif of Nm complex for structural studies using linker technique. A. Initial ITC experiment with wild type full length proteins such as CaM and Nm [19]. B. ITC experiment with optimized Minimum Binding Region (MBR) peptide of Nm and full length CaM. Optimized MBR from Nm mimics full length Nm in terms of binding with CaM. **C.** Comparison of SEC profile shows that the hydrodynamic radius of *apo-*CaM and *apo-*CaM-(Gly)_5_-NmIQ are same and the chimeric protein is a well folded complex. **D.** Crystal structure of *apo-*CaM-(Gly)_5_-NmIQ (PDB code: 4E53). CaM is shown in green and NmIQ is shown in magenta.

## PROCEDURE

1. ITC assay: Identification of MBR (minimum binding region) from Nm and Ng.
  1.1. Wildtype (WT) CaM, Nm and Ng are purified, as described previously [19].
  1.2. Prepare 10 µM of full length Nm/Ng and 150 µM of CaM in Buffer A (Recipes). The Schematic representation of the ITC experimental setup is depicted in **Figure 2A**.
  1.3. Degas all solutions for 10–20 min using a vacuum pump and centrifuge in order to avoid bubble formation in the sample cell during the experiment.
  1.4. Thoroughly wash the sample cell and the syringe using Buffer A. **NOTE:** Extensive washing of the cell and syringe is necessary to avoid any contaminants carried over from previous runs.
  1.5. Fill the reference cell with degassed buffer solution using a long-needle syringe.
  1.6. Fill the sample cell with 10 µM of Nm/Ng full-length protein, taking care to avoid the appearance of air bubbles in the cell. For the VP-ITC instrument, the net volume is ~1.5 ml.
  1.7. Fill the syringe with 150 µM of CaM solution according to the manufacturer’s recommendations. For the VP-ITC, the net volume is ~300 μl.
  1.8. Rinse off the excess ligand solution from the surface of the needle with water and carefully wipe the surface of the needle with a Kimwipe.
  1.9. Choose the instrument settings appropriate to your experiment. The conditions used in this case are: total number of injections (30 times), measurement temperature (25°C), reference power (15 μcal s^#x2012;^), initial injection delay (120 s), stirring speed (300 rpm), feedback mode gain (high feedback), injection volume (10 μl), duration of each injection (10 s), spacing between injections (240 s) and filter period (2 s).
**CRITICAL STEP**: The running parameters should be adjusted according to the recommendations of the ITC instrument manual. As the equilibration at low temperature is quite slow, we recommend 25°C as a starting point. Injection volume should be adjusted according to the ITC model. For the VP-ITC model, we suggest 5–10 μl. For more details, please see references [24,25].
  1.10. Start the experiment after gently setting the syringe in place. In the experiment discussed here, each run will take approximately 4 h. **TROUBLESHOOTING**
  1.11. For ITC experiments with CaM and IQ motif peptides, use 10 µM of CaM in the cell and 150 µM of Nm/Ng IQ peptides in the syringe.
  1.12. Dilute 1 mM peptide stock solutions with Buffer A (Recipes) to obtain a 150 µM final concentration for each peptide.
  1.13. Follow the steps 1.3-1.10. Rerun the ITC assay, filling the sample cell with CaM and syringe with different IQ peptide solutions. Maintain the same ITC running parameters as in the control titration.
  1.14. After the experiment, wash and maintain the ITC system according to the manufacturer’s recommendations.
2. DeepView analysis: Computational Analysis using known CaM-IQ motif complex.
  2.1. A step-by step tutorial for DeepView analysis is available at http://spdbv.vital-it.ch/TheMolecularLevel/SPVTut/ (**Fig. 2B**).
  2.2. At the pdb website (www.rcsb.org), search for the template model and download the pdb file. In the present case, we identified pdb 2IX7 as a template model to design the linked construct.
  **NOTE**: In some cases, a search for the PDB may need to be performed using the sequence of the protein of interest to identify the closest homolog complex, if available.
  2.3. Use the identified template pdb and modify the template (in this case, 2IX7) to generate the models for the selected interaction (in this case, *apo*-CaM-NmIQ and *apo*-CaM-NgIQ complexes). **TROUBLESHOOTING**
  2.4. Mutate the existing motif residues (in this case, myosin V IQ motif residues in the pdb 2IX7) with the sequence from MBR of the peptide of interest (in this case, Nm/Ng IQ motif) using the “MUTATE” operation.
  2.5. Use “ENERGY MINIMIZATION” to repair distorted geometries obtained due to mutating residues and for releasing internal constraints. Positions of the side-chains from the mutated residues can then be refined using this option.
  2.6. Once all residues are mutated based on the MBR of the peptide of interest (in this case, Nm/Ng IQ motif sequence), use the “SAVE” option to save the current model with an appropriate name, (in this case, “Model_Nm.pdb” or “Model_Ng.pdb”).
  2.7. Run Pymol and open the model (Model_Nm.pdb) and use the “Measurement” option to measure the distance between the selected terminus of the protein (C-terminal, CaM) and the terminus of the peptide (N-terminus, Nm). Repeat the same for other peptides (Ng).
  **NOTE**: This is an approximate way to determine the distance between the protein and peptides (CaM and Nm/Ng IQ motifs). Measurements obtained using this option provide only a linear distance. However, the model needs to be verified closely to determine the possible length of the linker required.
3. Fusion PCR and cloning: Linking MBR to structurally stable protein.
  3.1. Four different primers are then designed. In this case, P1- Forward primers for *CaM*. P2- Reverse primers for *CaM*, which incorporates DNA corresponding to (Gly)_5_ linker at the end of *CaM*. P3- Forward primers for *Nm/Ng IQ motifs*, incorporating base pairs that correspond to the (Gly)_5_ linker at the beginning of the *Nm/Ng IQ motifs*. P4- Reverse primers for *Nm/Ng IQ motifs*.
  3.2. Set up two types of PCR reactions. In this case, one to amplify *CaM* (with P1 and P2 primers) and another for each of the *Nm/Ng IQ motifs* (with P3 and P4 primers). Components are mixed on ice in 200 µl thin-wall PCR tubes.
  PCR reaction mix: 25 µl
  Template DNA was the clone which has been used to express individual proteins of CaM and Nm/Ng. We have used full-length Nm (accession no NP_032109) and Ng (accession no NP_071312) that were cloned into pQE30 (Qiagen, USA) plasmids and CaM (accession no NP_033920) that was cloned into MCS1 of pETDuet-1 vector (Novagen, USA).
  P1: 5` ACA CAT ATG GCT GAC CAA CTG 3`
  P2: 5` TCC TCC TCC TCC TCC CTT TGC TGT CAT 3`
  P3: 5` GGA GGA GGA GGA GGA GCT GCG ACC AAA 3`
  P4: 5` GAG AAG AAG GGT TGA CTC GAG ACA 3`
  3.3. PCR program for amplifying gene fragments
  3.4. Set-up a PCR reaction (25 µl) to fuse the two genes using genes amplified in the above step as template. Use the same PCR program described in step 3.3.

**Table:** 

Reagent	Amount per reaction
Template DNA	1 µl each of *CaM* and *IQ motif* amplified
Primers (P1 and P4)	300 nM final concentration
dNTPs	300 µM final concentration
KAPA HiFi DNA Polymerase	1 U
Buffer	as provided by the manufacturer
  3.5. Gene fusion is then verified using 1.5% agarose gel electrophoresis. PCR products obtained from step 3.4 should show a size corresponding to the sum of the two parts: in this case, *CaM* and *IQ motif* (**Fig. 2C**). **TROUBLESHOOTING**
  3.6. Purify the PCR product from the gel using the GeneAll Gel extraction kit.
  3.7. Double digest the purified PCR product (step 3.6) and the pGS21a vector (Genscript, USA) with *Nde*I and *Xho*I restriction enzymes and purify the double digests using the GeneAll PCR purification kit.
  3.8. Ligate the double digested PCR product and pGS21a vector using T4 DNA ligase. **TROUBLESHOOTING**
  3.9. Transform the ligated product into chemically competent *E. coli* DH5α cells using heat shock (Recipes).
  3.10. Inoculate colonies in 3 ml of LB broth and grow the culture at 37°C for 12–16 h. Perform a plasmid extraction using Qiagen mini prep kit.
  3.11. Verify the plasmids with DNA sequencing.
  3.12. Plasmids that contain the fused gene are then transformed into chemically competent *E. coli* BL21 cells using heat shock (Recipes).
4. Characterization of fused constructs: Purification and characterization of fused complexes (CaM-(Gly)_5_-NmIQ/NgIQ).
  4.1. For initial protein expression, inoculate a single colony in 100 ml of LB medium overnight at 37°C. Transfer the inoculum into 1 L of LB media (supplemented with 100 µg/mL ampicillin) and grow the culture at 37°C until the OD_600_ reaches between 0.6–0.8. The culture should then be maintained at 16°C before protein expression is induced with 0.15 mM IPTG. Cells are then grown for 16 h at 16°C. **NOTE:** Culture conditions, such as the IPTG concentration and temperature for IPTG induction may vary from protein to protein.
  4.2. For Single wavelength Anomalous Dispersion (SAD) phasing, seleno-L-methionine (SeMet) labeled proteins are produced using LeMaster media (Recipes). The same culture conditions as described in step 4.1 are used, with the exception that the LeMaster medium is used and the plasmids are transformed into DL41 cells (methionine auxotrophic strain).
  4.3. Cells from the 1 L culture are then collected by centrifugation at 9,000 *g* for 30 min using Avanti J-26 XP centrifuge (or similar). Resuspend the cell pellet obtained in 40 ml of lysis buffer (Recipes) in a 50 ml falcon tube. **HINT** Cell pellets obtained after centrifugation can be stored at -20°C for future use.
  4.4. Sonicate the cell suspension using 1 s ON/OFF pulses for 5 min. Centrifuge the cell lysate at 39,000 *g* for 30 min using Avanti J-26 XP centrifuge (or similar) to obtain a clear supernatant. **NOTE:** Make sure that the tip of the sonicator probe does not touch the sides and bottom of the falcon tube. Adjust the tube if an unusual loud noise is heard. **NOTE:** Make sure that the cell lysate is centrifuged to separate out the cell debris and the soluble protein fraction.
  4.5. Mix the supernatant with 5 ml of Ni-NTA resin that has been pre-equilibrated with lysis buffer and incubate for 1 h to allow binding. Wash the resin three times with lysis buffer without TritonX-100 (wash buffer) and elute the bound proteins using 10 ml of wash buffer supplemented with 500 mM imidazole. **NOTE** Make sure that the Ni-NTA resin is washed with water thoroughly and then equilibrated with lysis buffer.**TROUBLESHOOTING**
  4.6. Equilibrate a HiLoad 16/60 Superdex^TM^ 75 prep grade (GE Healthcare, Life Sciences) column with Buffer A (Recipes) by connecting it to an AKTA purifier. Use the following parameters on UNICORN 4.11 software: flow rate (1 ml/min), time (180 min) CV (column volume; 120 ml). The Schematic representation of size exclusion chromatography is depicted in **Figure 2D. NOTE:** Check all connections in the system to ensure that there are no leakages.
  4.7. Use a 5 ml loop for injecting the protein. Prior to injection, wash the loop twice with 5 ml of Buffer A. Run the sample according to the following parameters: flow rate (1 ml/min), column pressure limit (0.5 MPa), length of elution (1 CV), volume per fraction (1 ml). Start the program to elute the fusion protein in Buffer A. **CRITICAL STEP:** Carry out all the procedures of Ni-NTA chromatography, including this step, at 4°C in a cold room or inside a chromatography chamber. **CRITICAL STEP:** It is also important to perform SDS-PAGE on the peak fractions observed based on an absorbance at 280 nm from the size-exclusion chromatography. Peak fractions should contain only the protein of interest.**TROUBLESHOOTING**
  4.8. Use 3 kDa cut-off centricons to concentrate the eluted protein. Pool the fractions and spin it at 4,000 *g* at 4°C until the protein concentration is approximately 12 mg/ml as verified using Coomassie protein assay reagent (Recipes). **NOTE:** It is advisable to restrict centrifugation to 30 min and ensure that the concentration is checked. If necessary, Dynamic Light Scattering (DLS) can be performed to verify that concentrating the protein did not deteriorate its homogeneity.**TROUBLESHOOTING**
  4.9. Transfer the protein from the centricon to a 1.7 ml microfuge tube. Spin the sample at 14,000 *g* for 10 min at 4°C.
  4.10. Switch on the DLS machine and do not operate it until it reaches the set temperature (in this case 20°C). Prepare by washing the cuvette several times with water.
  4.11. Dispense 20 µl of water in the cuvette and place the cuvette into the appropriate position in the DLS machine as a blank control.
  4.12. Start the machine by clicking “start” (left panel). The counts for the blank should be less than 10 kilo counts/s (kCnt/s). Click “stop” (left panel) to stop the reading. **NOTE:** Extensive washing is required to avoid any interference from contaminants in previous runs.
  4.13. Remove the water and dispense 20 µl of concentrated protein into the cuvette. Click “start” (right panel), collect 20 readings and then click on “stop” (right panel). The schematic representation of dynamic light scattering is depicted in **Figure 2E. TROUBLESHOOTING**
  4.14. Once the readings are saved, remove the cuvette from the DLS machine, wash it with water and re-test the blank. Switch off the machine once the readings are taken for both the fused proteins.
5. Structure determination: Crystallization and structural studies of apo-CaM-(Gly)_5_-NmIQ and apo-CaM-(Gly)_5_-NgIQ.
  5.1. Crystallization trials are performed with 12 mg/ml of *apo-*CaM-(Gly)_5_-NmIQ and *apo*-CaM-(Gly)_5_-NgIQ using hanging drop vapor diffusion, as described previously [26]. The schematic representation of crystallization and structure determination is depicted in **Figure 2F. NOTE:** Do not disturb the crystallization trays frequently after setting up the drops.
  5.2. Initial crystals are obtained in a buffer solution consisting 0.2 M magnesium acetate tetrahydrate and 20% w/v polyethylene glycol 3350 (Hampton Research, cat. # HR2-126, condition # 25) for CaM-(Gly)_5_-Nm and 0.1 M HEPES sodium solution pH 7.5 and 1.4 M sodium citrate tribasic dehydrate (Hampton Research, cat. # HR2-110, condition # 38) for CaM-(Gly)_5_-Ng. **NOTE:** Crystallization conditions vary from case to case. **TROUBLESHOOTING**
  5.3. The quality of both crystals is improved by systematically varying the concentrations of the crystallization buffer components. Other additives (Hampton Research cat. # HR2-428) are also used [26]. **NOTE:** Concentrations should be varied gradually, as drastic variations in the concentrations of crystallization conditions may result in the loss of crystal formation.
  5.4. In this case, good-quality crystals are obtained in a buffer consisting of 0.12 mM magnesium acetate, 8% PEG 3350 and 10% ethanol for *apo-*CaM-(Gly)_5_-NmIQ and a buffer consisting of 0.1 M imidazole pH 8.0 and 1.2 M sodium citrate tribasic dehydrate for *apo*-CaM-(Gly)_5_-NgIQ.
  5.5. In this case, crystals are then tested using the in-house Rigaku Raxis V+. To test the crystals, single crystals are transferred to crystallization buffer supplemented with 10% glycerol using an appropriate size loop and soaked in cryoprotectant for 1 min. Using an appropriate sized loop, the crystals are then flash-cooled in N_2_ cold stream at 100 K.
  5.6. The following parameters are then used to test the diffraction quality of the crystal: distance to detector (60 mm), oscillation width (0.5°/image), oscillation range (1°), exposure time (30 sec/frame).
  5.7. The best crystals (diffract to 3 Å and with mosaicity < 1°) are then transferred and stored in liquid nitrogen storage cans. **CAUTION:** Wear cryoprotective gloves and a face mask when handing liquid nitrogen.
  5.8. Ship the crystals to the NSLS, Brookhaven National Laboratory, USA or the National Synchrotron Radiation Research Center (NSRRC), Taiwan, to collect complete SAD datasets for structure solution.
  5.9. Use the following parameters to collect complete datasets at SeMet peak wavelength (0.9795 Å) using the Quantum 4-CCD detector: distance to detector (300 mm) oscillation width (0.5°/image), oscillation range (360°), exposure time (2 s/frame). All datasets in this case were collected at 100 K.
  5.10. Process all the datasets using HKL2000 [27] and the .sca files generated are used for locating heavy atom (Se) positions, phasing and density modification using ShelxC/D/E [28] from CCP4. **NOTE:** It is important to follow the step-by-step procedure for scaling and phasing. CRTICAL STEP: Other programs, such as Phenix Autosol, can also be used for heavy atom (Se) phasing.
  5.11. Heavy atom locations obtained from ShelxC/D/E are then further used to autobuild, based on the sequence using Buccaneer [29] from CCP4. **NOTE:** Other programs, such as Phenix Autobuild, can also be used for sequence-based model building from heavy atom locations.
  5.12. Check the model obtained from the Buccaneer program in COOT [30]. Where necessary, the model can be manually built in COOT. **NOTE:** Build the model where significant electron density is available.
  5.13. In the present case, the crystal analysis revealed the presence of a twin fraction. Hence twin refinement was carried out during the refinement stage using Refmac5 [31].
  5.14. Stereochemistry of the models obtained are then verified using PROCHECK [32] in CCP4.
6. ITC assay: Validation.
  6.1. Closely examine the structures (in this case, those of *apo-*CaM-(Gly)_5_-NmIQ/NgIQ) to identify key interacting residues (in this case, from CaM and Nm/Ng IQ motif). **NOTE:** Use Contact run from CCP4 to identify the atoms involved in interactions and the distance between the interacting atoms (contacts <3.8 Å are considered for hydrophobic interactions and <3.2 Å are considered for H-bonding contacts).
  6.2. Site-directed mutagenesis of the interacting residues from the full-length protein can then be performed using Inverse PCR [33]. In this case, the mutations for CaM were D81A, E85A, F90A, E115A, E121A, M125A, F142A, E85A/F142A and F90A/E121A; for Nm, F42A, R43A and I46A; and for Ng, I33A, Q34A, F37A and R38A. Primers were designed as described in the paper by Dominy *et al.* [33]. **NOTE:** In this case, double mutations of CaM were obtained sequentially by performing the second mutation on the plasmid consisting of the first mutation.
  6.3. Use the following reaction and PCR program to generate the mutants.
PCR reaction mix: 50 µl reaction

**Table:** 

Reagent	Amount per reaction
Template DNA	10 ng
Primers (forward and reverse)	300 nM final concentration each
dNTPs	300 µM final concentration
KAPA HiFi DNA Polymerase	1 U
Buffer	as provided by the manufacturer
With the following program for PCR amplification:
  **Table:** 

Cycle number	Denature	Anneal	Extend
1	95°C, 2 min		
2 (16 times)	98°C, 20 s	52°C, 15 s	72°C, 3 min 30 s^b^
3			72°C, 5 min

^b^Change the time according to size, with extension time calculated based on 30 s kb^-1^ for the fragment being amplified.
  6.4. Verify the newly linear amplified plasmid using 1.5% agarose gel electrophoresis and purify the PCR product using GeneAll Gel extraction kit. **TROUBLESHOOTING**
  6.5. Transform the linear plasmid into chemically competent *E. coli* DH5α cells using heat shock (Recipes). Repeat steps 3.10–3.12 to obtain the mutants (in this case, the mutants for CaM, Ng and Nm).
  6.6. Purify the mutants (in this case, of CaM, Nm and Ng) as described previously [19].
  6.7. Prepare 10 µM of WT and mutants of full-length Nm/Ng in Buffer A to be used in the cell. Prepare 150 µM of WT and mutants of the full length protein (CaM) in Buffer A to be used in the syringe.
  6.8. Perform ITC experiments to compare the WT and mutant binding. In this case, for WT Nm/Ng against mutants of CaM and mutants of Nm/Ng against WT CaM.
  6.9. For ITC experiments, follow steps 1.2–1.10.
  6.10. Apart from ITC experiments, which can be used for validating any protein-protein complexes, we used *in vivo* electrophysiological experiments to validate *in vitro* findings. These *in vivo* experiments are specific to this particular study. For the details of the electrophysiological experiments, please refer to Kumar *et al.* [19].

## ANTICIPATED RESULTS

The Gly-linker strategy has been previously used to stabilize various protein complexes for structural studies [18]. We have adopted this procedure to determine the crystal structure of CaM in complex with two of its intrinsically unstructured binding partners, Nm and Ng, and further validated the findings *in vitro* and *in vivo* [19]. We identified the MBR of Nm and Ng that interacted with CaM using ITC experiments using various peptide lengths of Nm/Ng. We show that the MBR determined with ITC is comparable with the binding of full length Nm/Ng with CaM (**Fig. 3A** and **3B**). We employed known structures of CaM-IQ motif complexes to generate a template model of the CaM-Nm/Ng complex using DeepView, in which the IQ motifs of Nm/Ng interacted with the C-lobe of CaM. This is consistent with previous findings that Nm and Ng interacted with the C-lobe of CaM [34-36]. Thus, we linked the IQ motif of Nm/Ng to the C-terminus of CaM using a (Gly)_5_ linker. It is important to note that the distance between the two termini of the binding partners will vary depending upon the protein-protein complex being studied.

Expression of fused constructs avoids the need to purify individual proteins and it alleviates the inherent difficulties associated with generating a homogenous and stable complex. With our strategy, we observed homogeneous complexes of both Nm and Ng linked with CaM (Fig.3C). Subsequently, the linked complexes were crystallized and diffracted to 2.7 Å. The molecular replacement method did not yield the structure solution. Thus, SAD phasing was performed using seleno-L-Methionine labeled proteins (**ig. 3D**). Notably, the IQ motif of Ng and CaM adopted an intermolecular interaction [19]. This result demonstrates that if the linker length is not sufficient for the chimeric protein to engage in an intramolecular interaction, it will instead adopt an intermolecular interaction to stabilize the complex. This type of interaction has been observed previously for linked complexes [37].

It is important to validate the interactions obtained with linked binding partners. We performed this validation by mutating key residues involved in the interactions in the full length proteins, testing the binding *in vitro* with ITC and *in vivo* with electrophysiological experiments. In ITC experiments, substituting any key residue from Nm and Ng with alanine resulted in loss of interaction with CaM, while substituting any key residue from CaM with alanine resulted in reduced affinity towards Nm and Ng [19]. Furthermore, the *in vivo* experiments showed that mutating a key residue in Ng IQ motif resulted in its inability to potentiate synaptic transmission in CA1 hippocampal neurons [19]. Similarly, any appropriate *in vivo* experiments could be performed for any particular protein complex studied using this protocol. This protocol can be adopted to study other transiently interacting protein complexes involving structured or unstructured proteins, and offers potential application in studying the transient protein-protein interactions involved in various biological processes.
